# Identification of novel QTL contributing to barley yellow mosaic resistance in wild barley (*Hordeum vulgare* spp. *spontaneum*)

**DOI:** 10.1186/s12870-021-03321-x

**Published:** 2021-11-25

**Authors:** Yuhan Pan, Juan Zhu, Yi Hong, Mengna Zhang, Chao Lv, Baojian Guo, Huiquan Shen, Xiao Xu, Rugen Xu

**Affiliations:** 1 Key Laboratory of Plant Functional Genomics of the Ministry of Education / Jiangsu Key Laboratory of Crop Genomics and Molecular Breeding / Jiangsu Co-Innovation Center for Modern Production Technology of Grain Crops / Institutes of Agricultural Science and Technology Development, Yangzhou University, Yangzhou, 225009 Jiangsu China; 2 Jiangsu Institute for Seaside Agricultural Sciences and Yancheng Academy of Agricultural Science, Yancheng, 224002 Jiangsu China

**Keywords:** Wild barley (*Hordeum vulgare* spp. *spontaneum*), Barley yellow mosaic disease (BYMD), Quantitative trait loci (QTL), InDel markers

## Abstract

**Background:**

Barley yellow mosaic disease (BYMD) caused by *Barley yellow mosaic virus* (BaYMV) and *Barley mild mosaic virus* (BaMMV) seriously threatens the production of winter barley. Cultivating and promoting varieties that carry disease-resistant genes is one of the most powerful ways to minimize the disease’s effect on yield. However, as the BYMD virus mutates rapidly, resistance conferred by the two cloned *R* genes to the virus had been overcome by new virus strains. There is an urgent need for novel resistance genes in barley that convey sustainable resistance to newly emerging virus strains causing BYMD.

**Results:**

A doubled haploid (DH) population derived from a cross of SRY01 (BYMD resistant wild barley) and Gairdner (BYMD susceptible barley cultivar) was used to explore for QTL of resistance to BYMD in barley. A total of six quantitative trait loci (*qRYM-1H*, *qRYM-2Ha*, *qRYM-2Hb*, *qRYM-3H*, *qRYM-5H,* and *qRYM-7H*) related to BYMD resistance were detected, which were located on chromosomes 1H, 2H, 3H, 5H, and 7H. Both *qRYM-1H* and *qRYM-2Ha* were detected in all environments. *qRYM-1H* was found to be overlapped with *rym7,* a known *R* gene to the disease, whereas *qRYM-2Ha* is a novel QTL on chromosome 2H originated from SRY01, explaining phenotypic variation from 9.8 to 17.8%. The closely linked InDel markers for *qRYM-2Ha* were developed which could be used for marker-assisted selection in barley breeding. *qRYM-2Hb* and *qRYM-3H* were stable QTL for specific resistance to Yancheng and Yangzhou virus strains, respectively. *qRYM-5H* and *qRYM-7H* identified in Yangzhou were originated from Gairdner.

**Conclusions:**

Our work is focusing on a virus disease (barley yellow mosaic) of barley. It is the first report on BYMD-resistant QTL from wild barley accessions. One novel major QTL (*qRYM-2Ha*) for the resistance was detected. The consistently detected new genes will potentially serve as novel sources for achieving pre-breeding barley materials with resistance to BYMD.

**Supplementary Information:**

The online version contains supplementary material available at 10.1186/s12870-021-03321-x.

## Backgrounds

Barley (*Hordeum vulgare* spp. *vulgare*) is probably the earliest domesticated crop, which played an important role in the history of world agriculture [1]. Nowadays, barley is the fourth largest cereal crop in the world after maize, rice, and wheat [2]. For the last few decades, the soil-borne BYMD once seriously affected the yield of winter barley in the world [3–5]. In Europe, the prevalence of the disease result in up to 50% yield loss during each growing season [6, 7]. In China, a complete yield loss in barley has been recorded in the mid-1970s [8, 9].

BYMD belongs to soil-borne filamentous viruses transmitted by the vector *Polymyxa graminis*, caused by BaYMV and BaMMV [10, 11]. Before winter, the fungal vector invades the roots of barley seedlings and releases the virus before being transported from the roots to the leaves following water movement [12, 13]. Along with temperature rises in the next spring, the virus rapidly proliferates in the leaves of the susceptible varieties, bleaching chloroplasts and ultimately developed small, kite-shaped patches either pale green or yellow in appearance. Subsequently, the patches shape into chlorotic streaks, causing the death of older leaves, plant drawing, and panicle number reductions, ultimately decrease the grain yield [8]. The two viruses that cause BYMD are the positive-sense single-stranded RNA virus (‘+’-ssRNA virus), which belongs to the genus *Bymovirus* of the family *Potyvirdae*. The protein synthesis of the ‘+’-ssRNA virus is completely dependent on the translation machinery of the host cell, and the virus has a high mutation rate that quickly meets the needs of controlling the host cells [14]. The fungal vector carrying the BYMD virus can survive in the soil for more than 10 years and remain virulent [15]. It is difficult to control the disease by crop rotation or chemical application [16–19].

A total of 22 resistance genes to BYMD were mapped on seven chromosomes in barley. Among them, *rym4* and *rym5* were located on the terminal of chromosome 3H, encoding the eukaryotic translation initiation factor 4E (*eIF4E*), which were technically the first cloned resistance genes to BYMD. Another Four alleles of *eIF4E*, *rym6*, *rym10*, *rym*_*HOR4224*_, *rym*_*HOR3298*_, were identified in cultivated barley from East Asia [9]. Plant disease tolerance was obtained through an amino acid mutation in the coding region of eIF4E, which suppresses the binding with the viral genome-linked protein (VPg) protein of the virus and terminates the virus translation process [20, 21]. However, the resistance of *rym4* and *rym5* which were widely used in European breeding for years has been overcome by new virus strains [22, 23]. Another cloned resistance gene *rym1/11* from ‘Mokusekko 3’ was located on chromosome 4H, which belongs to the deletion mutation haplotype of the protein disulfide isomerase like 5-1 (*PDIL5-1*). The loss of function of *PDIL5-1* destroys the correct translation process of the virus in host plants, and the plants gain resistance [24]. The disease resistance of *rym1/11* has also been overcome by the new virus strains, and the variety which carries the gene independently showed mild susceptibility [25].

In addition to cloned *eIF4E* and *PDIL5-1*, *rym7* [26], *Rym16*^*HB*^ [27, 28], *Rym17* [29] and *Rym14*^*HB*^ [4] have been fine-mapped to a small region on chromosome 1H, 2H, 3H, and 6H, respectively. The remaining 10 BYMD-resistant genes *rym8* [30], *rym9* [31], *rym12* [32], *rym13* [33], *rym18* [29], *rym3* [34], *Un-designated* [35], *rym15* [36], *rym2* [32] and *rym7t* [37] have been preliminarily mapped.

Like many other crops, much genetic resource for biotic stress resistance in barley was lost during domestication, though wild barley is rich in genetic resources [38]. Once the disease-resistant genes are identified from wild barley, the breeding of resistance barley varieties can be accelerated by molecular marker assisted selection system [39]. For instance, resistance genes for barley powdery mildew [40, 41] and barley spot blotch were discovered from wild barley [42]. However, among all the resistance genes to BYMD, only *Rym14*^*HB*^ and *Rym16*^*HB*^ were derived from *Hordeum bulbosum* and others were derived from cultivated barley. In this study, novel QTL contributing to BYMD resistance was discovered from wild barley, which laid a foundation for the enrichment of yellow-mosaic resistance genetic resources and resistance breeding.

## Results

### Evaluation of resistance to BYMD in parents and DH populations

**Fig. 1:**
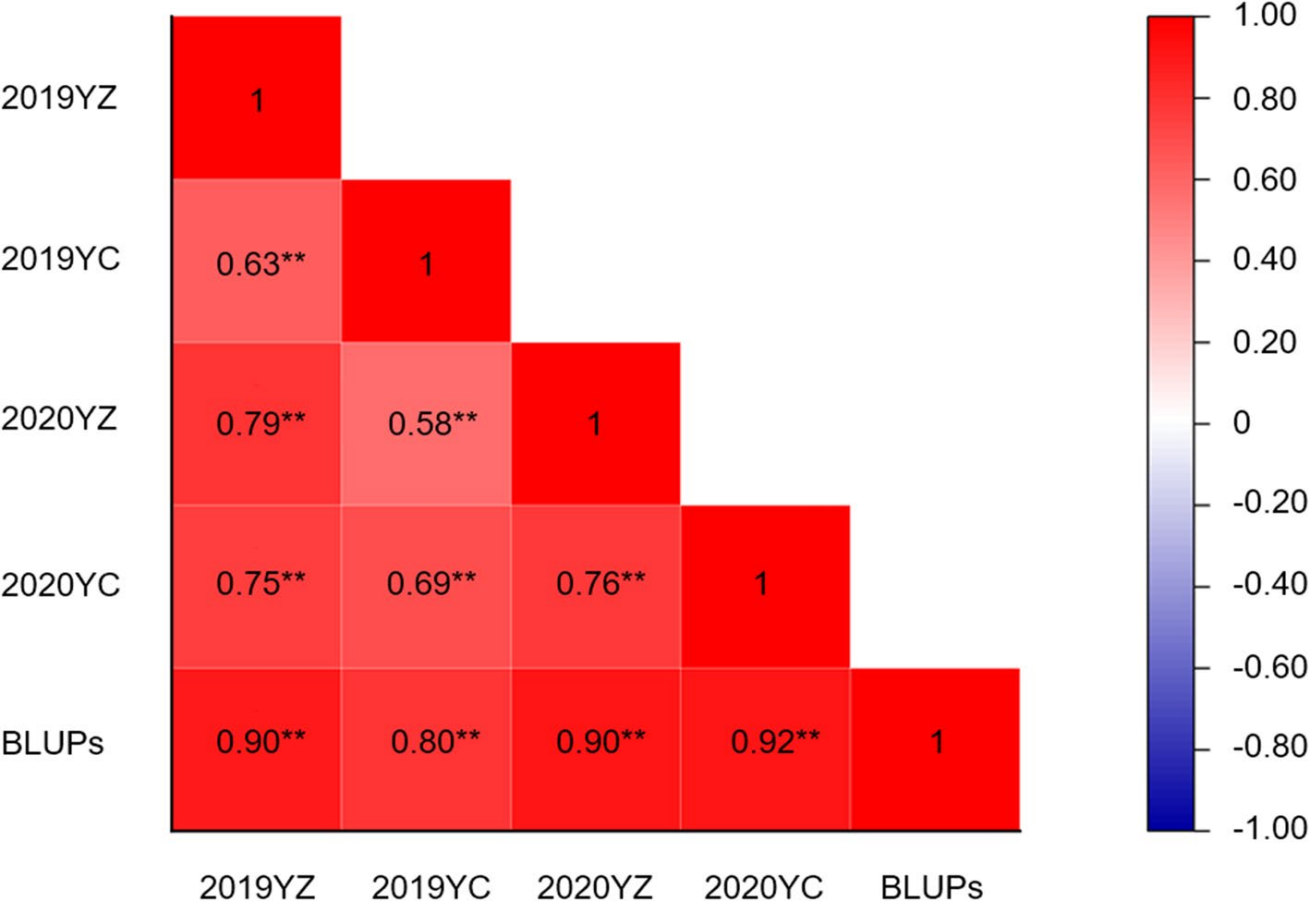
Correlation between the sAUDPS score in each environment and BLUPs

The standardized area under disease progress stairs (sAUDPS) and best linear unbiased predictions (BLUPs) of the resistant parent SRY01 in all environments were significantly lower than those of the susceptible parent Gairdner (Table 1 The sAUDPS score of BYMD in parents and DH population in each trial.and. Table S2. The disease grade of BYMD of parents and DH population in each investigation period). The analysis of variance (ANOVA) showed the effects of ‘Genotype’, those of ‘Genotype × Year’, as well as those of ‘Genotype × Site’ were reached to a significant level (. Table S3. ANOVA of sAUDPS score), indicating that the BYMD was not only controlled by genetic factors but also influenced by environmental factors represented by year and site. sAUDPS was significantly correlated between the environments and positively correlated with BLUPs (Fig. 1).

**Table 1:** The sAUDPS score of BYMD in parents and DH population in each trial

Trial	Mean of parents		DH lines		
	SRY01	Gairdner	Mean	Range	CV (%)
2019YZ	0.02	2.18	0.79	0.00-2.22	79.23%
2019YC	0.60	1.99	1.43	0.10-2.79	37.48%
2020YZ	0.27	2.55	0.90	0.00-2.22	86.53%
2020YC	0.44	2.49	1.25	0.00-2.53	59.71%
BLUPs	0.41	2.18	1.09	0.16-2.13	48.89%

**Fig. 2:**
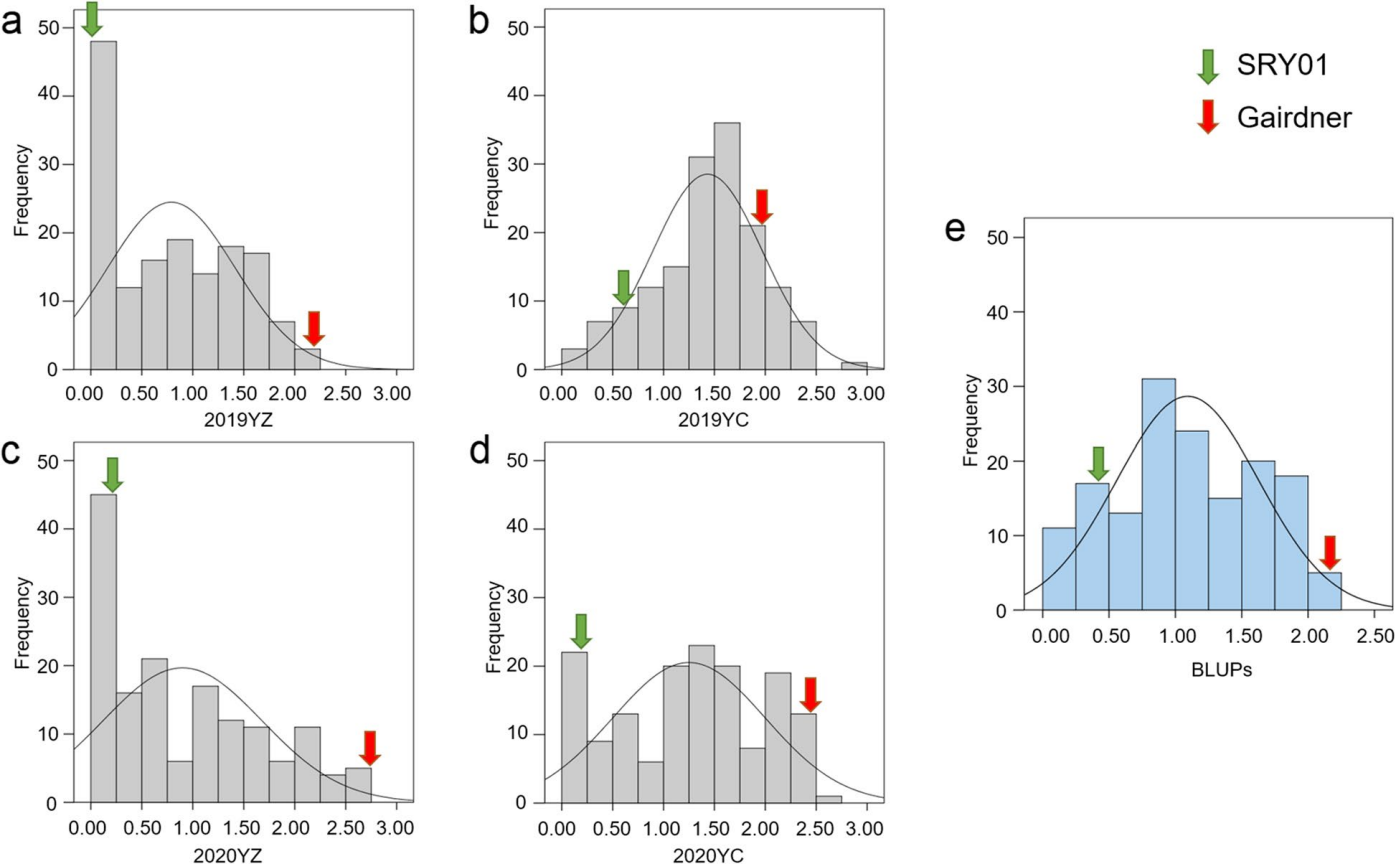
Frequency distribution of the sAUDPS score in each environment and BLUPs. **a**, Yangzhou sAUDPS score in 2019. **b**, Yancheng sAUDPS score in 2019. **c**, Yangzhou sAUDPS score in 2020. **d**, Yancheng sAUDPS score in 2020. **e**, BLUPs for all environments.

In the DH population, the sAUDPS score showed a continuous distribution (Fig. 2), indicating that BYMD was a quantitative trait controlled by multiple genes.

### Construction of linkage map and analysis of collinearity

After filtering the undesirable markers, 5210 high-quality SNP markers were used to construct a linkage map. The total length of the linkage map was 1007.07 cM (. Table S4. The distribution of single nucleotide polymorphism (SNP) markers on chromosomes of the DH population). We aligned the linkage map to the IBSC_v2 (International barley sequencing consortium) reference genome and found that there was good collinearity between the linkage map and the actual physical map except for chromosome 1H and 2H (Supplementary Material 5), laying the foundation for identifying candidate genes.

### QTL mapping

**Fig. 3:**
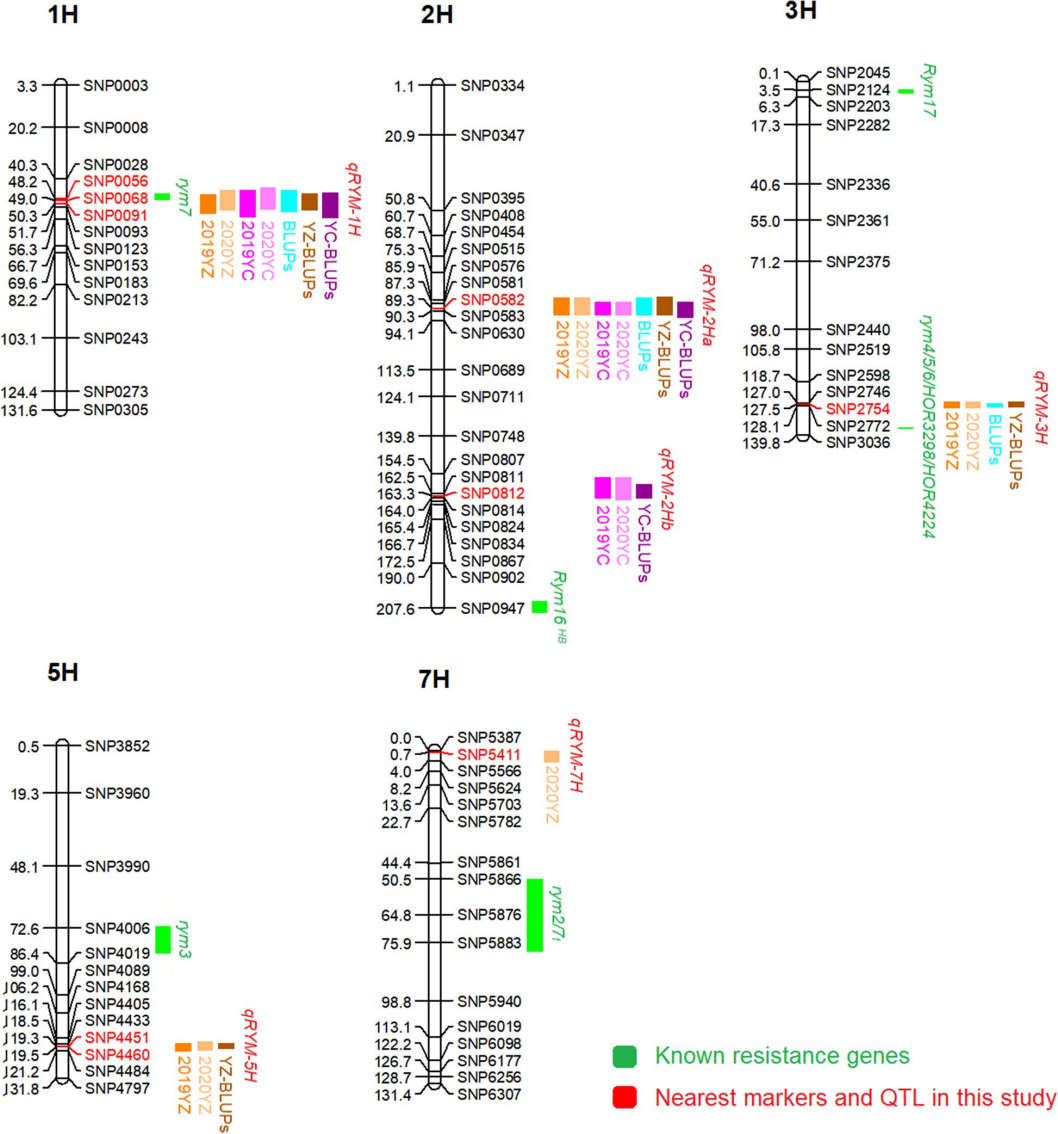
QTL for BYMD resistance identified from DH population and known BYMD-resistant genes

Six significant QTL have been identified when analyzing the sAUDPS score and BLUPs with the multiple QTL mapping (MQM) (Table 2 QTL for sAUDPS and BLUPs identified in the DH population). These QTL were mapped on chromosomes 1H (*qRYM-1H*), 2H (*qRYM-2Ha* and *qRYM-2Hb*), 3H (*qRYM-3H*), 5H (*qRYM-5H*), and 7H (*qRYM-7H*) (Fig. 3).

**Table 2:** QTL for sAUDPS and BLUPs identified in the DH population

QTL	Chr.	Trial	LOD	PVE (%)	Additive	QTL Interval	Nearest marker
*qRYM-1H*	1H	2019YZ	5.22	8.80	−0.19	46.22-53.74	SNP0068
		2020YZ	8.54	12.50	−0.28	44.72-52.39	SNP0068
		2019YC	6.89	14.40	−0.21	44.72-55.04	SNP0056
		2020YC	10.13	18.90	−0.34	42.15-50.28	SNP0056
		BLUPs	8.73	15.30	−0.21	44.72-53.08	SNP0068
		YZ-BLUPs	8.22	12.40	−0.21	46.22-52.39	SNP0068
		YC-BLUPs	11.08	19.80	−0.22	44.72-50.28	SNP0056
*qRYM-2Ha*	2H	2019YZ	7.08	12.30	−0.23	85.86-91.78	SNP0582
		2020YZ	8.28	12.00	−0.29	85.86-91.78	SNP0582
		2019YC	3.72	9.30	−0.17	87.28-91.78	SNP0583
		2020YC	7.55	16.40	−0.31	87.28-91.78	SNP0582
		BLUPs	9.99	17.80	−0.23	85.86-91.78	SNP0582
		YZ-BLUPs	7.97	11.90	−0.21	84.43-91.78	SNP0582
		YC-BLUPs	7.04	15.40	−0.19	87.28-92.99	SNP0582
*qRYM-2Hb*	2H	2019YC	9.09	20.90	−0.27	153.32-164.03	SNP0812
		2020YC	8.05	17.30	−0.34	154.55-165.45	SNP0812
		YC-BLUPs	10.63	22.10	−0.24	162.5-164.03	SNP0812
*qRYM-3H*	3H	2019YZ	4.45	7.50	−0.17	126.68-128.42	SNP2754
		2020YZ	4.10	5.60	−0.19	126.68-128.83	SNP2754
		BLUPs	4.92	8.10	−0.15	126.72-127.51	SNP2754
		YZ-BLUPs	5.10	7.30	−0.16	126.68-127.51	SNP2754
*qRYM-5H*^*G*^	5H	2019YZ	5.03	8.50	0.19	118.5-122.06	SNP4451
		2020YZ	4.84	6.70	0.21	118.08-121.56	SNP4460
		YZ-BLUPs	6.21	9.00	0.18	118.5-119.55	SNP4460
*qRYM-7H*^*G*^	7H	2020YZ	4.67	6.20	0.21	0.01-3.98	SNP5411

^G^According to the additive effect, QTL were from Gairdner. The others QTL were from SRY01

There was a stable QTL (*qRYM-1H*) on chromosome 1H, which was detected by the sAUDPS score and BLUPs in all environments, explaining phenotypic variation from 8.80 to 19.80%.

Two QTL were identified on chromosome 2H. *qRYM-2Ha* was identified on the middle of the chromosome which could be detected in all environments, explaining phenotypic variation from 9.30 to 17.80%. Near the end of chromosome 2H, there was a QTL *qRYM-2Hb* detected only in Yancheng (2019YC, 2020YC, and YC-BLUPs), and explained 17.30 to 22.10% of the phenotypic variation.

At the end of chromosome 3H, one minor QTL (*qRYM-3H*) was detected in Yangzhou (2019YZ, 2020YZ, and YZ-BLUPs) and BLUPs determined 5.60 to 8.10% of phenotypic variation.

The QTL *qRYM-5H* detected only in Yangzhou (2019YZ, 2020YZ, and YZ-BLUPs) was mapped on chromosome 5H, and *qRYM-7H* on chromosome 7H was detected only at 1 year in Yangzhou (2020YZ), explaining 9.00 and 6.20% of phenotypic variation respectively. And both of these two QTL were derived from the susceptible parent Gairdner.

### Sequencing of the cloned gene *eIF4E* on chromosome 3H

**Fig. 4:**
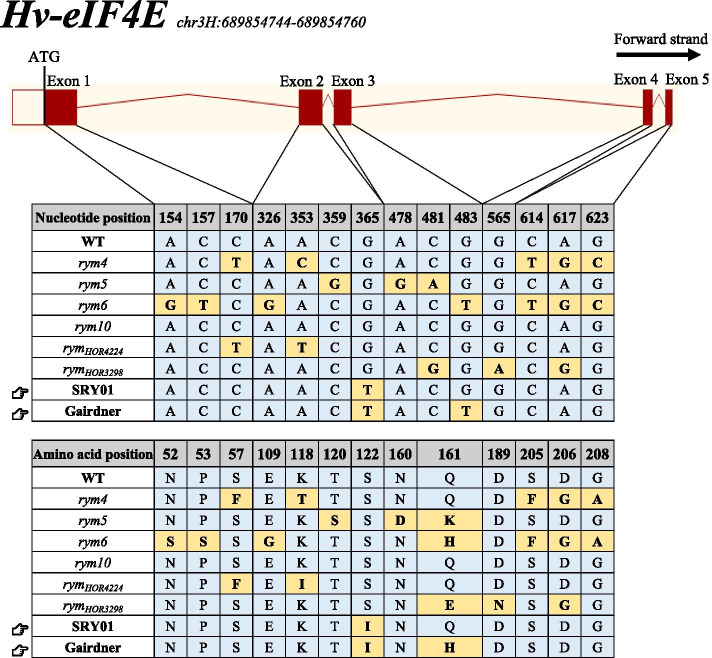
Haplotype analysis of the cloned gene *eIF4E* on chromosome 3H

Three primers covering the full CDS region of *eIF4E* were successfully used to amplify this gene from two parents. According to the sequencing results of *eIF4E*, there was only one coding mutation (G483T) on the third exon between the two parents (Fig. 4). According to the transcriptional information of *eIF4E*, this nonsynonymous mutation results in a Gln to His change at the amino acid level (Q161H). SRY01 belongs to haplotype L and Gairdner belongs to haplotype 36 of *eIF4E*. The two parents did not belong to the resistance haplotype of the cloned gene [9, 43].

### Developing of InDel markers

SRY01 and Gairdner were re-sequenced with a sequencing depth of 1.2×. In the physical interval of *qRYM-2Ha*, there were a total of 540 high-quality polymorphism difference loci between parents at the genomic level, including 451 SNPs and 89 InDels. In this interval, two practical InDel markers named 2H2745 and 2H1958 (. Table S6. Primer information for amplification of InDel in the interval of *qRYM-2Ha* on chromosome 2H) were successfully developed with polymorphism between parents (Supplementary Material 4).

## Discussion

### Effect of environmental factors on BYMD

The soil-borne BYMD is a serious threat to worldwide winter barley production [9]. The infection of the BYMD virus to plants is a dynamic process affected by environmental temperature [7]. In this study, ANOVA of the sAUDPS score showed that not only the interaction between genotype and site was significant, but also the site effect (Table S3. ANOVA of sAUDPS score), and the sAUDPS score of the DH population in Yangzhou for 2 years was lower than those in Yancheng (Table 1 The sAUDPS score of BYMD in parents and DH population in each trial.), indicating that the disease was more serious in Yancheng. Differences of BYMD virus strains between Yangzhou and Yancheng have been reported [7, 44]. Two strains of the BYMD virus were isolated in the Yancheng disease nursery, but only one in Yangzhou, and the coat protein and P2 fragments of the three strains were different [45]. In this study, *qRYM-2Hb* located on chromosome 2H was detected only in Yancheng in the 2 years by the sAUDPS score and BLUPs of Yancheng, indicating that *qRYM-2Hb* was stable resistance to the Yancheng virus strains specifically. Similarly, *qRYM-3H*, *qRYM-5H,* and *qRYM-7H* were detected only in Yangzhou. There is a co-evolutionary relationship between the BYMD virus and plant host, which seems to be an ‘arms race’ [9, 20, 21]. Isolate-specific QTL is an indispensable part of the formation of broad-spectrum resistance in resistant varieties [46], and it is helpful for plant hosts to obtain sustainable and comprehensive advantages in the race.

Moreover, the optimal temperature for BaYMV infection is 15 °C [17, 47], slightly higher temperature is beneficial for the infection, increasing virus replication and movement from roots to leaves [8, 48]. According to meteorological data, during the 5 months from barley sowing to the onset of BYMD, the monthly average temperature in Yangzhou in 2020 is higher than that in 2019 (. Table S5. Monthly average temperature of Yangzhou from 2018 to 2020). The higher temperature conditions in YZ2020 led to the aggravation of BYMD (Table 1 The sAUDPS score of BYMD in parents and DH population in each trial.). This might explain why *qRYM-7H* was only detected in YZ2020.

### A novel QTL contributing resistance to BYMD from wild barley

BYMD virus strains mutate rapidly, and the resistance of varieties (lines) carrying a single resistance gene can be easily overcome [31]. The varieties containing multiple resistance genes such as ‘Mokusekko 3’ (*rym1* and *rym5*) [49], ‘Chikurin Ibaraki 1’ (*rym15* and *Un-designated*) [35, 36] and ‘PK23-2’ (*Rym17* and *rym18*) [29] can resist most strains of BYMD, sustainably surviving from the disease in the nursery.

In this study, four resistance-related QTL were identified in SRY01, a wild barley with high resistance to BYMD in all environments. Among resistance-related QTL, there is no resistance-conferring gene to BYMD near *qRYM-2Ha* which was detected a total of 7 times by MQM in all environments. In summary, *qRYM-2Ha* is a novel major QTL related to BYMD resistance. The confidence interval of *qRYM-2Ha* is 85.856-91.781 cM in genetics, overlapping the physical interval of about 23.78 Mbp (613.472-637.249 Mbp in physics). According to the barley reference genome IBSC_v2, there are 242 annotated genes in this interval. After further searching the function of each gene on the BARLEX [50], nine annotated genes were found to be related to plant disease resistance (*.* Table S7. Genes related to resistance in the interval of *qRYM-2Ha* on chromosome 2H). Among them, three adjacent genes (*HORVU2Hr1G086860* starts at 625.19 Mbp, *HORVU2Hr1G087370* starts at 627.20 Mbp. and *HORVU2Hr1G087500* starts at 628.42 Mbp) were annotated as leucine-rich repeat receptor-like protein kinase family protein, which is associated with resistance proteins [51] which may present the candidate genes for *qRYM-2Ha*. At the genomic level, 89 indels were identified in the physical interval of *qRYM-2Ha* between parents. The development of these practical InDels markers such as 2H2745 and 2H1958 will lay the foundation for molecular marker-assisted selection breeding and the subsequent fine mapping.

### Comparison of QTL with known BYMD-resistant genes

Up to now, only one gene, *rym7*, has been mapped on chromosome 1H [7]. This gene was fine-mapped through linkage analysis based on 53 DH lines constructed from the disease-resistant variety HHOR3365 and the susceptible variety Igri. The phenotype was obtained by mechanical inoculation of the DH lines with the BaMMV strain, meanwhile, a linkage map constructed by combining 350 molecular markers specifically designed on chromosome 1H was used [26]. Bamg347, one of the nearest markers of *rym7*, had a physical location of 173.68 Mbp, which was closest to SNP0060 (172.96 Mbp in physics, 48.16 cM in genetics). SNP0060 was in the center of the confidence interval of *qRYM-1H* in our work, indicating there was an overlap between *rym7* and *qRYM-1H* (Fig. 3). We used different materials and methods to locate the resistance loci in the same region with previous researchers.

There is only one BYMD-resistant gene *Rym16*^*HB*^ delivered from *Hordeum bulbosum* was located on chromosome 2H, and its closest linkage marker was MWG949 (766.31 Mbp in IBSC or 664.04 Mbp in Morex v3.0) [27]. In this study, two QTL were mapped on chromosome 2H, and the nearest marker were SNP0582 (621.11 Mbp in in IBSC or 545.19 Mbp in Morex v3.0) and SNP0812 (723.86 Mbp in IBSC or 630.15 Mbp in Morex v3.0) respectively, which were far away from *Rym16*^*HB*^ (the minimum distance difference was about 42 Mbp in IBSC or 34 Mbp in Morex v3.0). Therefore, *qRYM-2Ha* and *qRYM-2Hb* on chromosome 2H identified in this study present new loci related to disease resistance.

*qRYM-3H* was located at the end of chromosome 3H, and its nearest marker SNP2754 (637.29 Mbp in physics) was close to the cloned gene *eIF4E* (689.85 Mbp in physics) by a distance of about 52 Mbp in physics or 10 cM in genetic position. To determine whether *qRYM-3H* is the cloned gene, the *eIF4E* of the two parents were sequenced and compared. The results showed that there was only one SNP between the parents, resulting in one amino acid change. Through the comparison of the *eIF4E* CDS sequence, the two parents did not belong to the haplotype of the known BYMD-resistant gene (Fig. 4) [43]. However, *qRYM-3H* was detected in Yangzhou and BLUPs, and its LOD value and contribution rate were low. It was speculated that *qRYM-3H* was a new minor locus that is only resistant to Yangzhou virus strains. In addition, two resistance-related QTL *qRYM-5H* and *qRYM-7H* from susceptible parent Gairdner on the chromosomes 5H and 7H were detected only in Yangzhou, both of which were far away from the known disease resistance genes (Fig. 3). Whether these two QTL with lower effects were related to disease resistance remains to be further studied.

## Conclusions

BYMD seriously threatens the production of winter barley. It is critical to identify novel resistance loci that can be used to genetically control BYMD. We used a DH population constructed from cultivated barley and wild barley as the material. After 2 years and two sites of resistance identification, combined with a high-resolution genetic map, we used the classical QTL mapping strategy to mine loci related to BYMD. Two major QTL for the resistance were identified. One of them was located in the same position to the known R gene *rym7*. Another one detected on chromosome 2H was not reported before and the resistance allele was from the wild barley parent. It is the first report on BYMD-resistant QTL from wild barley accessions (*Hordeum vulgare* spp. *spontaneum*). Based on biparental re-sequencing data, closely linked InDel markers for this novel major QTL were developed which could be used for MAS in barley breeding.

## Methods

### Plant materials and growth conditions

A total of 154 F_1_-derived doubled haploid (DH) lines generated from a cross between SRY01 and Gairdner were used in this study. The female parent SRY01 is a wild two-row barley (*Hordeum vulgare* spp. *spontaneum*) resistant to BYMD, and the male parent Gairdner is an Australian two-row malting barley (*Hordeum vulgare* spp. *vulgare*) susceptible to BYMD.

In the autumn of 2018 and 2019, the materials were sown in the BYMD nursery in Yangzhou University (Yangzhou, Jiangsu, China, 32° N, 119° E) and Jiangsu Coastal Agricultural Sciences Institute (Yancheng, Jiangsu, China, 33° N, 120° E) at the same time. Ten seeds of parental varieties and DH lines were sown in a 1.0 m row with 0.2 m of inter-row spacing and repeated three times separately.

### Evaluation of resistance to BYMD

The disease grade of the materials was qualitatively assessed from 0 to 4 [52]:

0, no visible symptom.

1, a small amount of pale-green chlorotic spots emerges, and the area of the yellow mosaic of new leaves is < 5%.

2, the number of chlorotic spots is more, the short-striped spots which parallel to the leaf veins appear, and the area of the yellow mosaic of new leaves is > 5% and < 25%.

3, the area of chlorotic spots expanded significantly and the short-striped spots developed into kite-shape patches, the area of the yellow mosaic of new leaves is > 25% and < 50% and the height of infected plant decreased slightly.

4, the leaves turn yellow, the plants dwarf or even wither, and the area of the yellow mosaic of new leaves is > 75%.

In the spring of 2019, four periods of investigation in Yangzhou were conducted on February 19, February 25, March 3, and March 10. Three periods of investigation in Yancheng were conducted on February 19, February 25, and March 5. In the spring of 2020, five periods of investigation in Yangzhou were conducted on February 9, February 18, February 24, March 4, and March 16. Five periods of investigation in Yangzhou were conducted on February 2, February 9, February 16, February 23, and March 1.

The disease grade data of the investigation was analyzed using Microsoft Excel 2019 software. The standardized area under disease progress stairs (sAUDPS) was used for resistance evaluation. This index can combine multiple observations of disease progress into a single value [53], and is not affected by the total investigation times. The index is calculated as follows:

$$\left(\mathrm{sAUDPS}=\right)\left\{\sum_{i=1}^{n-1}\left[\frac{y_i+{y}_{i+1}}{2}\times \left({t}_{i+1}-{t}_i\right)\right]+\left[\frac{y_1+{y}_n}{2}\times \frac{D}{n-1}\right]\right\}\times \frac{n-1}{Dn}$$

In this formula, the *n* is the total number of investigations, the *D* = *t*_*n*_ − *t*_1_ is the total investigation times, and the disease grade and date of the *i*-th investigation period are expressed by *y*_*i*_ and *t*_*i*_ respectively. The sAUDPS score of Yangzhou in 2019, Yangzhou in 2020, Yancheng in 2019, and Yancheng in 2020 are expressed by 2019YZ, 2020YZ, 2019YC, and 2020YC respectively.

The best linear unbiased prediction (BLUP) [54] Marker-assisted selection using best linear unbiased prediction on the sAUDPS score of the four environments were obtained using the lme4 package of the R [55]. In addition, BLUP was used to fit the phenotypes of the same location in different years, and the results were expressed by YZ-BLUPs and YC-BLUPs respectively. Use IBM SPSS Statistics 22 software for further descriptive statistics, ANOVA, and Pearson correlation analysis [56].

### Genotyping, map construction, and QTL mapping

DNA extraction and genotyping were performed as described in previous publications [57]. Two parental varieties and 154 DH lines were genotyped with DArTSeq (http://www.diversityarrays.com/). SNP markers were filtered according to the following criteria: heterozygous markers, markers without polymorphism between parents, and markers with a miss rate of more than 15% were eliminated. Referring to the methods of previous publications [58], the filtered SNP markers were used to construct a linkage map through MSTmap software [59]. The linkage map was aligned with the reference genome through OriginPro 9.1 software [60]. QTL mapping was conducted with MapQTL v6.0 software [61]. The naming of QTL follows the naming principle of McCouch et al. [62]. Linkage maps showing the QTL positions were drawn by using MapChart v2.32 software [63].

### Anchoring of known genes on the linkage map

To determine whether the QTL mapped in this study were the same as reported resistance genes of BYMD, sequence information of near linkage or flank markers of 22 reported resistance genes were collected. The physical positions of these markers in the barley IBSC reference genome and were obtained by the BLASTN (http://plants.ensembl.org/Hordeum_vulgare/Tools/Blast). The SNP markers which were closely linked to the reported disease resistance genes were anchored to the linkage map based on the physical location. Barley reference genome has been updated in 2021, and sequence information can be obtained online [64]. In order to more accurately determine the relationship between our two novel QTL and the physical location of known genes on chromosome 2H, BLASTN was performed in the Barley pseudomolecules Morex v3.0 reference genome (https://galaxy-web.ipk-gatersleben.de/).

### Sequencing of the cloned gene rym4

The cloned genes *rym4*, *rym5*, *rym6*, *rym10*, *rym*_*HOR4224*_, and *rym*_*HOR3298*_ belong to different haplotypes of *eIF4E* on chromosome 3H. Primers used for sequencing of *eIF4E* haplotypes were derived from previous publications (Table S1). The CDS region of *eIF4E* was amplified in SRY01 and Gairdner as referring to previous publications [9, 65, 66]. Then the amplified product was loaded on the agarose gel, and the bands which fit the product size were sent to BGI-Genomics (Shenzhen, China) for Sanger sequencing.

### Developing InDel markers from resequencing data

To serve resistance breeding, polymorphic molecular markers linked to resistance loci were developed. Economic low-depth resequencing was used to analyze the differences between parents at the genomic level. The DNA of the two parents were sent to Berry Genomics Co. Ltd. (Beijing, China) for resequencing by using the Illumina HiSeq-PE150 high-throughput sequencing platform. The Illumina clean reads provided by the sequencing company were mapped onto the assembled reference IBSC_v2 genome [67] with the Burrows-Wheeler Aligner [68] using the default parameters. The SAMtools mpileup program was used to assess variant sites [69]. TBtools software was used to extract sequence information near the variant sites [70]. InDel markers were designed using the Primer 3.0 (https://bioinfo.ut.ee/primer3-0.4.0/) according to the variant sites. The InDel markers were used to PCR-amplify DNA of both parental genotypes in a final volume of 10 μL, containing 40 ng genomic DNA, 0.2 μM forward and reverse primers, and 5 μL 2 × Taq Master Mix (Vazyme, Nanjing, China). Easy-to-operate PCR amplification was performed with 3 min at 95 °C for initial denaturation, followed by 34 cycles of 15 s at 95 °C, 20 s at 58 °C, 15 s at 72 °C, and finally one step of 10 min at 72 °C. The amplified product was detected in 4.0% agarose.

## Supplementary Information

**Additional file 1: Table S1.** Primer information for amplification of *HveIF4E.* **Table S2.** The disease grade of BYMD of parents and DH population in each investigation period. **Table S3.** ANOVA of sAUDPS score. **Table S4.** The distribution of single nucleotide polymorphism (SNP) markers on chromosomes of the DH population. **Table S5.** Monthly average temperature of Yangzhou from 2018 to 2020. **Table S6.** Primer information for amplification of InDel in the interval of *qRYM-2Ha* on chromosome 2H*.* **Table S7.** Genes related to resistance in the interval of *qRYM-2Ha* on chromosome 2H.

## Data Availability

The phenotype data of the DH population in this study are in Additional file 1 (Supplementary Material 1). The disease grade of BYMD of parents and DH population in each investigation period and ANOVA of sAUDPS score are in Supplementary. Table S2. The disease grade of BYMD of parents and DH population in each investigation period and Supplementary. Table S3. ANOVA of sAUDPS score, respectively. The genotype data including the physical and linkage map location of SNP markers are in Additional file 2 (Supplementary Material 2). The distribution of SNP markers on chromosomes of the DH population is listed in Supplementary. Table S4. The distribution of single nucleotide polymorphism (SNP) markers on chromosomes of the DH population. Primer information for amplification of *HveIF4E* and InDel in the interval of *qRYM-2Ha* on chromosome 2H is in Supplementary Table S6. Primer information for amplification of InDel in the interval of *qRYM-2Ha* on chromosome 2H, respectively. The sequences of the amplified *HveIF4E* of the two parents are listed in Additional file 3 (Supplementary Material 3, in FASTA formart). The InDel markers were used to amplify the genotype of the parents in agarose gel are shown in Additional file 4 (Supplementary Material 4). The collinearity of linkage map and the physical map was shown in Additional file 5 (Supplementary Material 5). The monthly average temperature of Yangzhou from 2018 to 2020 is listed in Supplementary. Table S5. Monthly average temperature of Yangzhou from 2018 to 2020 according to the meteorology database from http://www.nmc.cn/. Genes related to resistance in the interval of *qRYM-2Ha* on chromosome 2H are given in Supplementary. Table S7. Genes related to resistance in the interval of *qRYM-2Ha* on chromosome 2H, and the gene annotation information is accessible through BARLEYX online (https://apex.ipk-gatersleben.de/apex/f?p=284:10). The lme4 package of the R is available from https://cran.r-project.org/web/packages/lme4/index.html. The published barley reference genome is available from ftp://ftp.ensemblgenomes.org/pub/plants/release-48/fasta/hordeum_vulgare/dna/.

## Abbreviations

BYMD: Barley yellow mosaic disease
BaYMV: Barley yellow mosaic virus
BaMMV: Barley mild mosaic virus
DH: Doubled haploid
QTL: Quantitative trait loci
‘+’-ssRNA virus: Positive-sense single-stranded RNA virus
eIF4E: Eukaryotic translation initiation factor 4E
PDIL5-1: Protein disulfide isomerase like 5-1
sAUDPS: The standardized area under disease progress stairs
BLUP: Best linear unbiased prediction
BLUPs: Best linear unbiased predictions
IBSC: International barley sequencing consortium
MQM: Multiple QTL mapping
YZ: Yangzhou, China
YC: Yancheng, China
Gln: Glutamine
His: Histidine
ANOVA: Analysis of variance
