# Supplementary material for: Identification of novel QTL contributing to barley yellow mosaic resistance in wild barley (Hordeum vulgare spp. spontaneum)
Source: BMC Plant Biol. 2021 Nov 25;21:560. doi: 10.1186/s12870-021-03321-x (PMC8613928; doi:10.1186/s12870-021-03321-x)
Supplement: Supplementary file 1 — Additional file 1: Table S1. Primer information for amplification of HveIF4E. Table S2. The disease grade of BYMD of parents and DH population in each investigation period. Table S3. ANOVA of sAUDPS score. Table S4. The distribution of single nucleotide polymorphism (SNP) markers on chromosomes of the DH population. Table S5. Monthly average temperature of Yangzhou from 2018 to 2020. Table S6. Primer information for amplification of InDel in the interval of qRYM-2Ha on chromosome 2H. Table S7. Genes related to resistance in the interval of qRYM-2Ha on chromosome 2H. [file 12870_2021_3321_MOESM1_ESM.zip › Supplementary Table 2.docx]

Table S 2 The disease grade of BYMD of parents and DH population in each investigation period

| Year | Site | Period | Parents | |  | DH lines | | |
| --- | --- | --- | --- | --- | --- | --- | --- | --- |
|  |  |  | SRY01 | Gairdner |  | Average | Range | CV (%) |
| 2019 | Yangzhou | 2/19 | 0.00 | 1.64 |  | 0.63 | 0.00-2.53 | 98.00% |
|  |  | 2/25 | 0.00 | 2.07 |  | 0.82 | 0.00-2.67 | 83.00% |
|  |  | 3/3 | 0.00 | 2.82 |  | 1.07 | 0.00-2.60 | 74.00% |
|  |  | 3/10 | 0.09 | 2.16 |  | 0.63 | 0.00-1.80 | 81.00% |
|  | Yancheng | 2/19 | 0.79 | 1.88 |  | 1.52 | 0.33-2.73 | 38.00% |
|  |  | 2/25 | 0.52 | 2.07 |  | 1.45 | 0.00-2.67 | 40.00% |
|  |  | 3/5 | 0.52 | 2.00 |  | 1.34 | 0.00-3.00 | 47.00% |
| 2020 | Yangzhou | 2/9 | 0.00 | 2.11 |  | 0.35 | 0.00-2.67 | 183.00% |
|  |  | 2/18 | 0.00 | 2.33 |  | 0.50 | 0.00-2.67 | 147.00% |
|  |  | 2/24 | 0.67 | 2.33 |  | 1.10 | 0.00-3.00 | 84.00% |
|  |  | 3/4 | 0.44 | 2.78 |  | 1.23 | 0.00-3.33 | 83.00% |
|  |  | 3/16 | 0.22 | 3.00 |  | 1.15 | 0.00-3.33 | 81.00% |
|  | Yancheng | 2/2 | 0.11 | 1.44 |  | 0.26 | 0.00-1.33 | 138.00% |
|  |  | 2/9 | 0.11 | 2.00 |  | 0.76 | 0.00-2.67 | 96.00% |
|  |  | 2/16 | 0.33 | 3.00 |  | 1.63 | 0.00-3.00 | 61.00% |
|  |  | 2/23 | 0.22 | 3.00 |  | 1.55 | 0.00-3.00 | 67.00% |
|  |  | 3/1 | 1.44 | 3.00 |  | 2.04 | 0.00-3.00 | 50.00% |
